# Real-Life Experience of Molnupiravir in Hospitalized Patients Who Developed SARS-CoV2-Infection: Preliminary Results from CORACLE Registry

**DOI:** 10.3390/antibiotics11111541

**Published:** 2022-11-03

**Authors:** Tommaso Lupia, Silvia Corcione, Nour Shbaklo, Lucio Boglione, Stefano Torresan, Simone Mornese Pinna, Barbara Rizzello, Roberta Bosio, Valentina Fornari, Maria Teresa Brusa, Silvio Borrè, Francesco Giuseppe De Rosa

**Affiliations:** 1 Unit of Infectious Diseases, Cardinal Massaia, 14100 Asti, Italy; 2 Department of Medical Sciences, Infectious Diseases, University of Turin, 10126 Turin, Italy; 3 School of Medicine, Tufts University, Boston, MA 02111, USA; 4 Department of Translational Medicine (DiMET), University of Eastern Piedmont, Via Solaroli 17, 28100 Novara, Italy; 5 Unit of Infectious Diseases, Saint Andrea Hospital, 13100 Vercelli, Italy

**Keywords:** molnupiravir, SARS-CoV-2, oral antiviral, COVID-19

## Abstract

Real-life experience of molnupiravir treatment is lacking, especially in people hospitalized for underlying diseases not related to COVID-19. We conducted a retrospective analysis regarding molnupiravir therapy in patients with SARS-CoV-2 infection admitted for underlying diseases not associated with COVID-19. Forty-four patients were included. The median age was 79 years (interquartile range [IQR]: 51–93 years), and most males were 57,4%. The median Charlson Comorbidity Index and 4C score were, respectively, 5 (IQR: 3–10) and 9.9 (IQR: 4–12). Moreover, 77.5% of the patients had at least two doses of the anti-SARS-CoV-2 vaccine, although 10.6% had not received any SARS-CoV-2 vaccine. Frequent comorbidities were cardiovascular diseases (68.1%), and diabetes (31.9%), and most admissions were for the acute chronic heart (20.4%) or liver (8.5%) failure. After molnupiravir started, 8 (18.1%) patients developed acute respiratory failure, and five (11.4%) patients died during hospitalisation. Moreover, molnupiravir treatment does not result in a statistically significant change in laboratory markers except for an increase in the monocyte count (*p* = 0.048, Z = 1.978). Molnupiravir treatment in our analysis was safe and well tolerated. In addition, no patients’ characteristics were found significantly related to hospital mortality or an increase in oxygen support. The efficacy of the molecule remains controversial in large clinical studies, and further studies, including larger populations, are required to fill the gap in this issue.

## 1. Introduction

Molnupiravir (also known as MK-4882) is an oral antiviral pro-drug that reduces the transmission of SARS-CoV-2 in infected patients [1]. This molecule, a ribonucleoside of N-hydroxycytidine (NHC), mutates viral RNA and inhibits viral replication [1,2]. It was initially studied to treat the Influenza virus in 2019 [1]. 

During the SARS-CoV-2 pandemic, molnupiravir was administered to animal species, and it showed the possibility to prevent viral transmission and SARS-CoV-2 inhibition [2]. In-vitro experiments demonstrated similar results [3].

Consequently, studies on its use in humans were performed to evaluate the drug’s safety and efficacy. According to the MOVe-OUT Phase 3 trial results, molnupiravir provides an approximately 50% reduction in death or the need for hospitalization on day 29 when administrated at a dose of 800 mg twice a day for five days [4].

The Data Safety and Monitoring Board recommended that the clinical study assessing molnupiravir be terminated early after approximately half of the sample enrolled. As a result of the apparent advantage of the medicine over the placebo, the clinical trial was terminated, and all other participating sites ended enrollment within about a week [5,6]. Approximately 90% of the planned sample had been recruited, and follow-up data were available at the time of discontinuation of the experiment [5,6]. 

The drug is currently not authorized in the European Union, but it can be used in emergency settings [7]. In Italy, it was administered beginning on 5 January 2022. It is recommended to use molnupiravir early after a SARS-CoV-2 diagnosis, within five days of symptoms in patients who do not require supplemental oxygen but have an increased risk of developing severe COVID-19 [8]. Nausea, dizziness, headache, and diarrhoea are the common side effects of the treatment. Other side effects include back-pain influenza-like syndrome [8]. It is not recommended during pregnancy and in women who are likely to be pregnant [8]. It could also have a teratogenic effect on sperm cells [8].

We report here a real-life experience in the use of molnupiravir for the treatment of patients with SARS-CoV-2 infection, admitted for other diseases and without evidence of viral pneumonia or need for oxygen support, barring oxygen for previous underlying diseases.

## 2. Results

From 5 January to 5 March 2022, the medical records of 45 patients were extracted from the CORACLE registry; 44 had complete data on in-hospital follow-up and mortality and were included in the present analysis. Included patients were from Cardinal Massaia Hospital in Asti (*n* = 16), S. Andrea Hospital in Vercelli (*n* = 12) and the City of Health and Sciences University Hospital (Molinette Hospital) in Turin (*n* = 16), Italy. The patients’ demographic and clinical characteristics are shown in Table 1.

The median age at admission was 79 years (interquartile range [IQR]: 51–93 years) and *n* = 17 (42.6%) of the patients were female. Of the sample, the median Charlson Comorbidity Index was 5 (IQR range: 3–10). The most frequent comorbidities were cardiovascular disease *n* = 32 (68.1%, including a history of hypertension, cardiac failure and myocardial infarction), *n* = 15 (31.9%) diabetes mellitus, *n* = 10 neurological diseases (21.3%) and *n* = 9 solid organ tumour (19.1%). The 4C mortality score for COVID-19 was performed; the median was 9.9 (IQR range: 4–12). Moreover, 83% of the patients had undergone a completed course of vaccination (2 doses); of those, 66% had received a booster dose. Of the patients treated with molnupiravir, 10.6% had not received any dose of the anti-SARS-CoV-2 vaccine, 6.4% had previously contracted COVID-19, 90,6% were admitted to medical wards and 9.4% were admitted to surgical wards. The most frequent reasons of admission were heart failure (20.4%) and liver failure (8.5%) (Table 1).

The time interval from the last dose of vaccine and the first in-hospital SARS-CoV-2 positive nasopharyngeal polymerase chain reaction (PCR) swab was 96.8 (IQR range: 7–240) days. During hospitalisation, after molnupiravir administration was begun, 8 (18.1%) patients developed mild or moderate acute respiratory failure. One patient needed high-flow oxygen and underwent remdesivir antiviral therapy and steroid treatment. 

None developed new symptoms or pneumonia at chest X-ray during molnupiravir treatment. Five (11.4%) patients died during hospitalisation due to underlying disease. The median age of the deceased patients was 68 years (IQR: 60–76); the median age of the patients who were discharged was 76 years (IQR: 63–89). Five (10.5%) patients developed bacterial infection during hospitalisation: two patients developed urinary tract infections (both from penicillin-sensible Escherichia coli) and two from vancomycin-resistant Enterococcus faecium infections.

Moreover, molnupiravir treatment did not result in a statistically significant change in creatinine, AST, ALT, WBC and RDW, except for a mild increase in the monocyte count (*p* = 0.048, Z = 1.978) (Table 2). 

Patients’ characteristics were not significantly related to hospital mortality or the need to start oxygen therapy (Table 3 and Table 4). Patients who died clinical features, were summarized in Table 5.

## 3. Discussion

As of April 2022, more than two years after the outbreak of the disease, approximately 14.8 million Italians have been infected and 159,000 have died because of SARS-CoV-2 [9]. The majority of those afflicted (about 65%) were under the age of 50 [9].

We described a cohort of high comorbid hospitalized patients for underlying diseases not related to COVID-19 who suffered from hospital-acquired SARS-CoV-2 infection and were treated with molnupiravir.

In our case series, the median age of patients infected with SARS-CoV-2 and treated with molnupiravir was higher than the median age of infected outpatients in the period of study (January–March 2022). This difference could be related to the higher median age of hospitalised patients compared to outpatients. Moreover, accordingly to other Italian real-life study regarding molnupiravir age distribution was similar to our retrospective analysis [10,11] Due to immunosenescence and additional comorbidities, older patients may be at a greater risk of death and complications related to SARS-CoV-2 infection and the prescription of molnupiravir was initially focused on this population [12].

The high burden resulting from comorbid conditions in this population was evidenced by the median CCI (*n* = 5), and the distribution of comorbidities was in line with previously reported data from the CORACLE registry, in which cardiovascular diseases (68.1% vs. 81%, respectively) and diabetes mellitus (31.9% vs. 21%, respectively) were the most frequent comorbidities [13]. De Vito and colleagues reported, in an Italian real-life experience with molnupiravir, as a first comorbidity cardiovascular disease (50.0%), however the second most frequent pathology were chronic lung disease (29.2%), instead diabetes is in fifth place (21.4%) [11].

From T0 to T5 of molnupiravir treatment we did not observe any changes in WBC, RBC, platelets, lymphocytes, eosinophils. GOT, GPT, creatinine nor renal or liver functions. Of note, in this small series, we described a significant reduction in monocyte count during molnupiravir treatment. We speculated that this reduction in monocytes could be theoretically related to a reduction in SARS-CoV-2 viral replication and perhaps immune activation.

In fact, in the lungs of COVID-19 patients, the most abundant immune cells are monocytes and macrophages, and these cells appear to play an important role in disease pathogenesis [12]. In individuals with mild or moderate COVID-19, monocytes in the blood show an inflammatory, interferon-stimulated gene (ISG)-driven phenotype, while in those with severe symptoms, cellular dysfunction is the primary hallmark, including the loss of HLA-DR expression and an increase in S100 alarmin expression [14,15]. Pro-inflammatory cytokines and cytotoxic effector cells are recruited to the site of infection by hyperactivated pulmonary macrophages from invading monocytes, resulting in exacerbated tissue damage at the site of infection [14,15].

From a clinical point of view, most patients remained paucisymptomatic, with no need to initiate oxygen therapy even if patients were hospitalized due to underlying diseases not related to COVID-19 and therefore the population analysed was slightly different what has recently been described in the literature. In fact, molnupiravir was created as a drug intended to treat patients in an outpatient setting as early treatment.

As reported in clinical trials [3], molnupiravir was well tolerated in our cohort of patients despite the fact that it was administered in a population with a high average age, with multiple comorbidities. Oral administration, despite the pill burden, was optimal; all patients completed the prescribed treatment course without skipping a dose. The treatment plans were easy for health care workers to manage, as they did not need to resort to the use of more invasive routes of administration. Being made up of pills, the drug is also easy to store without the need for low temperatures.

In the MOVE-IN study, a randomised, placebo-controlled, double-blind phase 2/3 trial, a 5-day course of molnupiravir up to 800 mg twice daily in a hospitalised population was not associated with clinical benefits [16]. The MOVE-IN study was conducted at 65 hospitals in 15 countries globally [16]. The lack of clinical benefits in this population was thought to be related to delayed treatment initiation in relation to the temporal pattern of COVID-19 symptom onset and illness severity [16]. In contrast, almost all the patients in the present study resolved COVID-related symptoms as a result of molnupiravir treatment.

In the univariate analysis we did not find any clinical characteristics or comorbidity associated with a higher risk of death or risk of respiratory failure. Despite that we found that aged, high comorbid patients resulted in a high survival, low risk of respiratory failure after molnupiravir therapy. We have confirmed these favourable findings also in the non-vaccinated population and in patients with two doses of anti-SARS-CoV-2 vaccine. Favourable outcomes in our results are in line with real-life data published by other centres [10,11,17,18], despite that most of the patients considered in other studies were treated from the beginning as outpatients. Patients’ characteristics who died in the study populations were reported in Table 5: we found high comorbid patients, with high Charlson comorbidity index who died for complications other than COVID-19.

Moreover, in our study we did not find any differences in terms of mortality or risk of progression to respiratory failure between vaccinated or unvaccinated patients treated with molnupiravir. Recently Khoo et al., published data regarding the effect of molnupiravir on virological response by SARS-CoV-2 variant and in vaccinated patients [19]. In this study viral load reductions during molnupiravir treatment were observed in both vaccinated and unvaccinated individuals [19]. In addition, Khoo and colleagues stated that no participants in the molnupiravir group were hospitalized [19]. Interestingly Wong and colleagues in their real-life use of molnupiravir showed in the subgroup analyses of study outcomes stratified by age and vaccination status, results comparing molnupiravir use and non-use were significant for reduced risks of all-cause mortality and in-hospital disease progression among patients older than 60 years [17]. At this time vaccination status before molnupiravir treatment seems to be protective especially in patients over 60 years old, but this data are not conclusive. This study has several limitations. Foremost is the limited sample size. Another limitation that must be addressed by future research is the role that vaccination rate plays on the outcomes of the population and the risk related to body mass index. The majority of molnupiravir treatment studies have been performed on unvaccinated patients. In our cohort, most of the patients had completed the vaccination cycle. As reported in the literature, the vaccine is effective at reducing mortality but especially at reducing the severity of the disease in patients. This did not allow us to highlight statistically significant elements in relation, for example, to the time of negative infection or outcome. Surely, with the implementation of related data, it will be possible to run more precise re-evaluations in future research.

## 4. Conclusions

In conclusion, this is the first real-life experience regarding clinical outcomes, safety and tolerability of molnupiravir in a SARS-CoV-2 infected population of high comorbid patients, with a high number of vaccinated patients hospitalized for underlying diseases not related to COVID-19. In our brief report molnupiravir treatment does not result in a statistically significant change in laboratory markers, except for a mild increase in the monocyte count and we did not define patients’ characteristics significantly related to hospital mortality or the need to start oxygen therapy. Favourable outcomes in our results are in line with real-life data published in the literature, despite that most of the patients considered in other studies were treated from the beginning as outpatients. Despite that, The efficacy of the molecule remains controversial in large clinical studies, and further studies, including larger populations, are required to fill the gap in this issue.

## 5. Materials and Methods

We conducted a retrospective study to evaluate describe the use of molnupiravir treatment in patients with SARS-CoV-2 infection, admitted for underlying diseases not related to COVID-19. In addition, we have described adverse effect and safety of molnupiravir treatment in our population. Moreover, we performed a literature search to evaluate the pharmacological and clinical characteristics related to the use of molnupiravir in SARS-CoV-2 infection. 

We enrolled patients hospitalised at Cardinal Massaia Hospital in Asti and the City of Health and Sciences University Hospital (Molinette Hospital) in Turin, within the CORACLE study register in Piedmont, Italy [13,20,21]. The following inclusion criteria were applied: adults over the age of 18 who developed hospital-acquired COVID-19 five days after admission with a negative SARS-CoV-2 antigenic and/or molecular swab and symptomatology compatible with SARS-CoV-2 according to European Medicines Agency (EMA) criteria [6]. As previously mentioned, the primary endpoint was to establish the efficacy and safety of molnupiravir. We determined the patients’ demographic and anamnestic data (age, sex and comorbidities using the Charlson Comorbidity Index score) and vaccine status. We focused on the onset of COVID-19-related symptoms other than the initial symptoms during treatment and if any patients needed an increased oxygen demand (in acute or organ liver transplantation; OLT). Periodically, on the first day (T0) and the last day (T5) of therapy blood tests were performed to obtain data on creatinine, aspartate transaminase (AST), alanine aminotransferase (ALT), sodium (Na), potassium (K) and cells blood count (CBC). We also reported if an additional anti-SARS-CoV-2 therapy (i.e., remdesivir, tocilizumab or dexamethasone) was necessary during the treatment and if there were any adverse effects. Follow up was performed within 2 weeks after the end of the treatment regimen.

### Statistical Analysis

A database was created using a Microsoft Excel table for data collection. We performed statistical analysis using SPSS^®^ software. Data was entered and analysed using SPSS^®^ version 27.0 (IBM^®^). Descriptive analysis was reported as frequencies and percentages for categorical variables and means and standard deviations for numeric variables. Dichotomous variables were evaluated against mortality or need for oxygenation using Chi square test. Continuous variables were tested for normality by Kolmogorov Smirnov test. Normally distributed variables were evaluated using t-tests. Not normally distributed variables were evaluated using Mann-Whitney test. Statistical significance was defined as less than 0.05. The role of real-life safety of molnupiravir treatment on blood count (e.g., white cell blood count -WBC-, red cell blood count -RBC-, platelets, lymphocytes, monocytes and eosinophils) and liver (e.g., glutamic oxaloacetic transaminase—GOT- and serum glutamic pyruvic transaminase -GPT- ) and kidney (e.g., creatinine) function at start of molnupinavir (T0) and at the end of therapy (T5) was analysed using the Wilcoxon ranked test. Comorbidities such as kidney, pulmonary, oncologic, hematologic and and neurologic diseases were around the quarter of the total patients. Therefore, they weren’t accounted for when testing association with mortality or necessity of oxygenation.

## 6. Patents

This section is not mandatory but may be added if there are patents resulting from the work reported in this manuscript.

## Figures and Tables

**Table 1:** Main Characteristics of Hospitalized Patients Treated With Molnupiravir.

Main Characteristics of Hospitalized Patients Treated with Molnupiravir *n* = 44 (100%)	
Age (median, years)	79 (IQR range, 51–93) o <50 yo (*n* = 0) o 50–64 (4) o 65–79 (16) 80–94 (24)
Sex	Male 27 (57.4)
Past Smoker	4 (8.5)
BMI	21.5 (IQR range, 18.7–30.1)
*Scores*	
CCI	5 (IQR range, 3–10)
4C	9.9 (IQR range, 4–12)
*Comorbidities*	
Cardiovascular diseases	32 (68.1)
CKD	14 (29.8)
Pulmonary diseases	12 (25.5)
Neurologic diseases	10 (21.3)
Solid tumor	9 (19.1)
Haematological diseases	5 (10.6)
Diabetes	15 (31.9)
SOT	1 (2.1)
Rheumatologic diseases	2 (4.5)
*Reason of Admission in Hospital*	
Heart failure	9 (20.4)
Liver failure	4 (8.5)
Anemia	3 (6.8)
Femoral neck fracture	3 (6.4)
CAP	2 (4.3)
Gastrointestinal Diseases	3 (6.4)
Neurological Diseases	2 (4.5)
Cancer	2 (4.5)
UTI	3 (6.4)
*Previous COVID-19 or SARS-CoV-2 Vaccination*	
Previous COVID-19	3 (6.4%)
V0	5 (10.6)
V1	0
V2	8 (17)
V3	31 (66)
First doses	39 (30 BioNTech/Pfizer; 8 Moderna; 1 Johnson and Johnson)
Second doses	39 (31 BioNTech/Pfizer; 8 Moderna)
Third Doses	31 (24 BioNTech/Pfizer; 7 Moderna)
*Previous Oxygen for Chronic O2-therapynd Start Oxygen after SARS-CoV*-	
Patients in O2-therapy before molnupiravir	8 (18.1)
Start O2 during molnupiravir or increase	8 (18.1)
*Hospital acquired infections*	
Nosocomial superinfections	5 (10.5)
UTI	4 (*Escherichia coli* ESBL, *Proteus mirabilis*, *Enterococcus faecium* Vancomycin-resistant; *Klebsiella oxytoca*)
BSI	1 (*Enterococcus faecium* Vancomycin-resistant)
*Events during molnupiravir Treatment*	
Adverse events during Molnupiravir	0 (0)
Pill missed	0 (0)
*Outcomes*	
Survival	39 (88.6)
*Time to positivity and SARS-CoV-2 positivity*	
Time from last vaccination to SARS-CoV-2 positivity	96.8 (7–240) days
Time from admission to SARS-CoV-2 S/S start	15 (5–33) days

Abbreviations: IQR: interquartile range; CCI: Charlson comorbidity index; 4C: four “C” score; CKD: chronic kidney disease; SOT: solid organ transplant; CAP: community acquired pneumonia; UTI: urinary tract infection; V: vaccine; O2: oxygen; BMI: body mass index; UTI: urinary tract infection; BSI: blood-stream infection; ESBL: extended-spectrum Beta-lactamases; S/S: signs or symptoms.

**Table 2:** Laboratory values changes between Time from Day 0 (T0) and Time from Day Five (T5) in patients treated with Molnupiravir.

Laboratory Value	T0	T5	Wilcoxon Test *
AST (UI/L)	49.88 (9–296)	41.3 (15–157)	0.737
ALT (UI/L)	36.12 (3–254)	29.02 (9–110)	0.522
Creatinine (mg/dL)	1.05 (0.38–2.5)	0.97 (0.39–1.9)	0.128
WBC (total count)	6676 (2500–18,930)	9201 (3120–65,330)	0.501
Lymphocyte (absolute count)	1149 (340–2450)	3500 (510–57,490)	0.081
Monocyte (absolute count)	639 (270–1410)	546.67 (230–580)	0.048
Eosinophile (absolute count)	150(0–560)	127(0–810)	0.394
PLTS (absolute count)	189(0.112–400)	221 (0.243–479)	0.162

Abbreviations: AST: aspartate aminotransferase; ALT: alanine aminotransferase; WBC: white blood cell; PLTS: platelets; T0: time from Day 0; T5: time from day 5. * Data were entered and analysed using SPSS^®^ version 27.0 (IBM^®^).

**Table 3:** Patients’ characteristics distributions between dead and survived patients.

Variable	Survived *n* = 39 (88.6%)	Dead *n* = 5 (11.4%)	Total *n* = 44 (100%)	*p*-Value
*Basal characteristics*				
Gender (Males)	16 (36.4%)	1 (2.3%)	17 (38.6%)	0.36
Age	76 ± 13 (SD)	68 ± 8 (SD)	79 ± 13 (SD)	0.06
*Clinical characteristics*				
Cardiovascular disease/hypertension	29 (65.9%)	3 (6.8%)	32 (72.7%)	0.49
Pulmonary disease	9 (20.5%)	3 (6.8%)	12 (27.3%)	0.08
4C score	9.8 ± 2.03 (SD)	11 ± 1.4 (SD)	10 ± 2 (SD)	0.43
CCI	5.9 ± 1.8 (SD)	5.5 ± 1.8 (SD)	6 ± 2 (SD)	0.57
*Vaccination*				
Not vaccinated	4 (9.1%)	1 (2.3%)	5 (11.4%)	0.51
Vaccinated (Second dose)	8 (18.2%)	0	8 (18.2%)	0.26
Vaccinated (Third dose)	27 (61.4%)	4 (9.1%)	31 (70.5%)	0.61

Abbreviations: CCI: Charlson comorbidity index; 4C: four “C” score; SD: standard deviation.

**Table 4:** Patients’ characteristics distributions between patients that need oxygen after molnupiravir start and patients without need of oxygen start.

Variable	No Oxygenation *n* = 36 (81.8%)	Oxygenation *n* = 8 (18.1%)	Total *n* = 44 (100%)	*p*-Value
*Basal characteristics*				
Gender (Males)	14 (31.8%)	3 (6.8%)	17 (38.6%)	0.94
Age	75 ± 12.5 (SD)	79 ± 11 (SD)	76 ± 13 (SD)	0.36
Past smoker	2 (4.5%)	2 (4.5%)	4 (9.1%)	0.08
*Clinical characteristics*				
Cardiovascular disease/hypertension	28 (63.6%)	4 (9.1%)	32 (72.7%)	0.11
Kidney disease	12 (27.3%)	2 (4.5%)	14 (31.8%)	0.64
Pulmonary disease	10 (22.7%)	2 (4.5%)	12 (27.3%)	0.87
Neurologic disease	8 (18.2%)	2 (4.5%)	10 (22.7%)	0.86
Oncologic disease	6 (13.6%)	3 (6.8%)	9 (20.5%)	0.18
Hematological disease	5 (11.4%)	0	5 (11.4%)	0.26
Diabetes	13 (29.5%)	2 (4.5%)	15 (34.1%)	0.54
Nosocomial infection	4 (9.1%)	1 (2.3%)	5 (11.4%)	0.74
4C score	9.8 ± 2 (SD)	10 ± 2 (SD)	10 ± 2 (SD)	0.55
CCI	6 ± 1.9 (SD)	6 ± 1.9 (SD)	6 ± 2 (SD)	0.35
*Vaccination*				
Not vaccinated	3 (6.8%)	2 (4.5%)	5 (11.4%)	0.17
Vaccinated (Second dose)	7 (21.9%)	1 (3.1%)	8 (25%)	0.77
Vaccinated (Third dose)	26 (59.1%)	5 (11.4%)	31 (70.5%)	0.58

Abbreviations: CCI: Charlson comorbidity index; SD: standard deviation; 4C: four “C” score.

**Table 5:** Patients’ characteristics of deceases patients.

Age/Sex	CCI	4C	Hospital Admission	Other Comorbidities	Vaccination Status	Start O2	Superinfections	COVID-19 Related Death
63/F	7	7	Chronic hepatic failure with increasing ascitis	COPD, CNS	Three doses	No	No	No
60/M	8	7	Urinary Obstruction and urinary tract stenting	CV, COPD, Malignancy, DM	Three doses	Yes, NC	Yes, septic shock	No
59/F	8	9	Chronic kidney failure in hemodyalisis with refractory hyperkalemia	CV, COPD, DM	Three doses	No	No	No
74/F	11	10	Atrial fibrillation and myocardial injury	CV, CKD, DM	Unvaccinated	Yes, NC	No	No
82/F	12	11	Pace-maker device infection	CV, CKD, COPD, DM	Three doses	No	No	No

Abbreviations: CCI: Charlson comorbidity index; 4C: four “C” score; CV: cardiovascular; COPD: chronic obstructive pulmonary disease; CNS central nervous system; DM: diabetes mellitus; CKD: chronic kidney disease; F: female; M: male; NC: nasal canula.

## Data Availability

Not applicable.
